# Preclinical Evidence of *Withania somnifera* and *Cordyceps* spp.: Neuroprotective Properties for the Management of Alzheimer’s Disease

**DOI:** 10.3390/ijms26115403

**Published:** 2025-06-04

**Authors:** Gabriele Tancreda, Silvia Ravera, Isabella Panfoli

**Affiliations:** 1 Department of Health Sciences (DISSAL), University of Genoa, 16132 Genoa, Italy; 2 Department of Experimental Medicine, University of Genoa, 16132 Genoa, Italy; 3 IRCCS Ospedale Policlinico San Martino, 16132 Genova, Italy; 4 Department of Pharmacy (DIFAR), University of Genoa, 16132 Genoa, Italy

**Keywords:** antioxidant effect, cordycepin, neuroinflammation, neuroprotection, oxidative stress, phytotherapy, withanolides

## Abstract

Alzheimer’s disease (AD) is considered one of the main pathologies of our time, whose incidence and prevalence are suggested to be strongly underestimated. AD presents as a complex neurodegenerative condition characterized by marked neuroinflammation and a significant decline in the cognitive and mnemonic functions of affected patients. Recognized AD pathological hallmarks include amyloid beta plaque and neurofibrillary tangle formation, synaptic dysfunction with considerable apoptosis of cholinergic and dopaminergic neurons, and high levels of oxidative stress and neuroinflammation. The available pharmacological treatments are represented by acetylcholinesterase inhibitors to treat the mild to moderate form of the disease and N-methyl-D-aspartate inhibitors alone or in combination with the previously cited ones in the late stage of the neurodegenerative condition. Furthermore, emerging drug therapies such as monoclonal antibodies are promising agents in AD management. Although scientific evidence highlights these chemicals as effective in slowing down disease progression, significant limitations behind their employment derive from the notable dose-dependent side effects and the single-target mechanism of action. In this context, two well-studied phytotherapeutics, *W. somnifera* (*W. somnifera)* and fungi belonging to the genus *Cordyceps*, have gained attention for their chemical composition regarding their neuroprotective and anti-inflammatory effects. Ashwagandha (obtained principally from the roots of *W. somnifera*) is an adaptogen that relieves stress and anxiety. It contains several ergostane-type steroidal lactones—such as withanolides and withaferin A—and various alkaloids, contributing to its antioxidant and neuroprotective effects. Likewise, cordycepin is the main bioactive principle found in *Cordyceps fungi*. This natural nucleoside has been reported to possess therapeutic potential as an anti-cancer, immunomodulatory, and anti-inflammatory agent, with some studies suggesting a beneficial role in AD treatment. The purpose of the present review is to investigate the pharmacological properties of *W. somnifera* and *Cordyceps species* in the context of AD treatment and explore the therapeutic potential of the constitutive bioactive molecules in preclinical models mimicking this neurodegenerative condition.

## 1. Alzheimer’s Disease: Pathophysiology and Current Therapies

Alzheimer’s disease (AD) is a complex, multifactorial, neurodegenerative disease that presents itself as a global public health problem and the most common cause of dementia, representing 60–70% of total cases. Globally, the incidence of this condition is expected to triple, profoundly affecting the quality of life of patients and their respective caregivers [1].

AD’s classical pathological hallmarks include the deposition of amyloid β (Aβ) peptides that aggregate, forming extracellular neuritic plaques localized throughout the cerebral cortex. The generation of these peptides depends on the cleavage and processing of the amyloid precursor protein (APP), an integral membrane protein physiologically present in the synapses of neurons. Oligomers of Aβ, especially Aβ_42_, exert synaptotoxic effects through interaction with different receptors, leading to calcium influx, oxidative stress, mitochondrial dysfunction, and neurotoxicity [2]. Another key neuropathological hallmark of AD is the hyperphosphorylation of the tau protein, a microtubule-associated protein in axons, synaptic compartments, and the nuclear membrane. Soluble forms of tau are highly toxic and responsible for shattering mitochondrial function, calcium homeostasis, and impairing synaptic transmission and plasticity. Under pathological conditions, tau tends to become hyperphosphorylated and detach from microtubules, undergoing transmission through trans-synaptic and non-synaptic routes. It accumulates in neurons and aggregates into neurofibrillary tangles (NFTs), neuropil threads, and dystrophic neurites, promoting neurotoxicity and the progression of the cognitive decline characteristics of AD [3]. In this context, the intra- and extra-neuronal landscape alteration promotes glia-mediated neuroinflammation. Microglia and astrocyte cells shift from their physiological supportive role and drive neuroinflammatory pathways, accelerating neuronal death. Aβ and tau aggregates chronically overstimulate microglial receptors Trigger Receptor Expressed on Myeloid Cells-2 (TREM2) and C-X3-C Motif Chemokine Receptor 1 (CX3CR1), triggering NOD-like receptor protein 3/Apoptosis-associated speck-like protein containing a CARD (NLRP3/ASC) inflammasome assembly with the consequent caspase-1 activation and release of pro-inflammatory cytokines, such as IL-1β, TNF-α, and IL-6, which, in turn, amplify neuroinflammation, contributing to neuronal dysfunction and neurodegeneration. In detail, the release of pro-inflammatory cytokines triggers the apoptosis pathway, increases neuronal excitotoxicity and immune activation, and causes overproduction of oxidative stress, leading to neuron death [4]. Additionally, in a self-perpetuating loop, these cytokines promote the activation of p38 mitogen-activated protein kinase (p38 MAPK) and glycogen synthase kinase-3 Beta (GSK-3β), which exacerbates further tau hyperphosphorylation and aggregation [5].

Current drug therapies approved by the Food and Drug Administration (FDA) aim to manage symptoms of cognitive decline, although most are helpful only in the mild to moderate initial phases of AD. Acetylcholinesterase (AChE) inhibitors, N-methyl-D-aspartic acid receptor (NMDAR) antagonists, and anti-amyloid monoclonal antibodies represent the principal class of medications administered in clinical practice today. Donepezil, Galantamine, and Rivastigmine enhance cholinergic transmission and operate by increasing acetylcholine (ACh) levels. Memantine, differently, is approved for the moderate to severe forms of AD, alone or in combination with an AChE inhibitor, and acts as an antagonist of NMDARs. By suppressing excessive glutamatergic activity, Memantine can reduce Ca^2^-mediated excitotoxicity, ultimately preventing neuronal damage. Anti-amyloid monoclonal antibodies (e.g., Aducanumab and Lecanemab) represent another possible treatment. This class of therapeutics promotes Aβ plaque removal and clearance through three complementary pathways: the microglia-activated phagocytosis and lysosomal degradation of pathological peptide aggregates, the direct binding of antibodies to Aβ fibrils in the central nervous system (CNS), and the peripheral “sink” effect that favors the efflux of Aβ from brain interstitial fluid into the blood [6]. Given the broad impact of this neurodegenerative disorder, the prevention and management of AD symptoms through safe and effective bioactive compounds and complementary strategies should be highly considered. In this regard, several phytotherapeutics have attracted attention for their potential contribution to ameliorating neuroinflammation, metabolic status, and clinical markers specific to AD. Examples are represented by *Panax ginseng*, *Crocus sativus*, *Curcuma longa*, and *Berberis*, which have all been proven to exert beneficial effects in AD thanks to their recognized anti-inflammatory, antioxidant, and anti-apoptotic properties [7].

The present work aims to systematically review current literature evidence about the potential pharmacological properties of phytotherapeutics *Withania somnifera* (*W. somnifera*) and *Cordyceps species* (*C.* spp.) in preclinical models (rodents) of AD, enabling us to report and evaluate possible clinical applications.

## 2. *W. somnifera* and *C.* spp.: Bioactive Components and Major Mechanisms of Action

*W. somnifera* and the medical fungi *C.* spp. are both herbal drugs with long traditional use and clinical application in Asian medicine cultures.

### 2.1. W. somnifera: Pharmacological Profile and Therapeutic Targets

**Figure 1:**
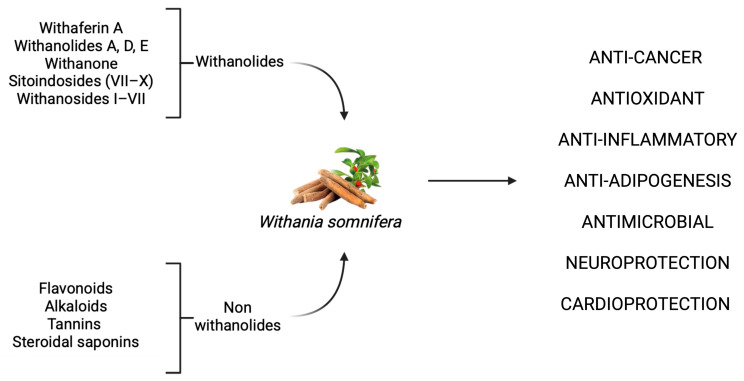
The principal bioactive constituents and major biological effects and properties of *W. somnifera* extract. The scheme shows the principal bioactive compounds present in *W. somnifera* (on the **left**) and the principal cellular effects (on the **right**). The figure was created in BioRender. Gabriele Tancreda. (2025) https://app.biorender.com/illustrations/681b34276dcbff013d2f6f4b (accessed on 2 June 2025).

*W. somnifera*, commonly known as Indian ginseng or Ashwagandha, is a geographically widespread herb. It grows spontaneously in arid and semi-arid zones ranging from the European basin to southern Africa and the Middle East, and spreading through Central and South Asia across India, Pakistan, and Bangladesh. The botanical component of interest with pharmacological applications is the raw root, whose integration has been associated with cardioprotective, anti-cancer, sedative, antimicrobial, and neuroprotective benefits [8]. *W. somnifera,* belonging to the Ayurveda tradition, has attracted growing attention due to the health benefits derived from its consumption [9]. Preparations in the form of root powder have been traditionally used as a tonic and adaptogen to enhance vitality and counteract fatigue, anxiety, and insomnia while promoting longevity and memory functions. Nonetheless, *W. somnifera* has also been used in pastes and decoctions, and to produce topical oils which have been employed for the management of respiratory, dermatological, and inflammatory conditions [8]. In detail, the roots and leaves of *W. somnifera* contain a diverse array of phytochemicals with multi-target activity, including over 300 identified C-28 steroidal lactones known as withanolides, as well as other non-withanolide constituents such as flavonoids, tannins, and steroidal saponins. Key bioactive compounds found in the root extract include withaferin A, withanolides A, D, and E, withanone, sitoindosides (VII–X), withanosides I–VII, and various alkaloids such as somniferine and pseudotropine. These highly pleiotropic compounds exert potent activities across a wide range of disease models, including anti-cancer, antiproliferative, radiosensitizer, anti-inflammatory, anti-adipogenesis, antimicrobial, and many more (Figure 1) [10,11,12,13].

#### 2.1.1. *W. somnifera* as a Promising Anti-Cancer Agent: Mechanistic Evidence Across Tumor Models

Regarding the anti-cancer effect, *W. somnifera* compounds have demonstrated significant anti-cancer properties across various cancer types, including breast, colon, prostate, ovarian, gastric, and melanoma. These effects are mediated through multiple molecular mechanisms that target key pathways involved in cancer cell proliferation, survival, metastasis, and angiogenesis [14], influencing several transcription factors involved in cancer aggressiveness and progression such as nuclear factor kappa-light-chain-enhancer of activated B cells (NF-κB), nuclear factor erythroid 2–related factor 2 (Nrf2), and p53 [9,13].

In breast cancer models, withaferin A induces G2/M phase cell cycle arrest and apoptosis by modulating cyclin-dependent kinases and increasing ROS production, leading to mitochondrial dysfunction and activation of the intrinsic apoptotic pathway [15]. Specifically, withaferin A downregulates anti-apoptotic proteins, such as Bcl-2, and upregulates pro-apoptotic proteins, like Bax, resulting in caspase activation and PARP cleavage [14]. Additionally, withaferin A inhibits the STAT3 signaling pathway, which is often constitutively active in triple-negative breast cancer cells, thereby reducing cell proliferation and metastasis [16].

In colon cancer cell lines (HCT-116, SW-480, and SW-620), withaferin A suppresses the Notch-1 signaling pathway and downregulates the Akt/NF-κB/Bcl-2 axis, leading to apoptosis [13]. It also induces G2/M cell cycle arrest by disrupting the spindle assembly checkpoint through the degradation of Mad2 and Cdc20 proteins [17]. The pharmacological activity of withaferin A was also studied in HCT116 cell xenograft tumors in nude mice, where this compound attenuated tumor growth [18].

Similarly, in an in vitro model of human cervical cancer, withaferin A administration was responsible for strong cell proliferation inhibition, downregulation of oncoproteins expression, and an increase in p53 and p21 levels [19].

In melanoma models, withaferin A induces apoptosis through ROS-mediated mitochondrial pathways and downregulation of anti-apoptotic proteins. This leads to DNA fragmentation and cell death in melanoma cells [20].

Additionally, withaferin A exhibits anti-angiogenic and anti-metastatic properties by targeting multiple pathways. It inhibits angiogenesis by downregulating vascular endothelial growth factor (VEGF) expression and interfering with the ubiquitin–proteasome pathway in endothelial cells [14]. Moreover, withaferin A suppresses metastasis by inhibiting epithelial-to-mesenchymal transition (EMT) markers such as vimentin and N-cadherin and by reducing matrix metalloproteinases (MMPs) involved in extracellular matrix degradation [14].

#### 2.1.2. Anti-Inflammatory Mechanisms of *W. somnifera*: Modulation of NF-κB, Nrf2, and Cytokine Pathways

Several studies have demonstrated the anti-inflammatory properties of *W. somnifera* (Ashwagandha) to be exerted by bioactive compounds such as withanolides, sitoindosides, withanosides, and various alkaloids. One of the key molecular mechanisms involves the inhibition of the nuclear factor kappa B (NF-κB) signaling pathway, a critical regulator of inflammation. Withaferin A blocks the activity of IκB kinase β (IKKβ), thereby preventing the degradation of IκBα and subsequent nuclear translocation of NF-κB. This leads to decreased transcription of pro-inflammatory cytokines, including TNF-α, IL-1β, and IL-6, as well as pro-inflammatory enzymes such as COX-2 and iNOS [21,22,23].

Simultaneously, *W. somnifera* activates the Nrf2 pathway, enhancing the expression of antioxidant enzymes like heme oxygenase-1 (HO-1), superoxide dismutase (SOD), and glutathione peroxidase (GPx), which counter oxidative stress—a major driver of chronic inflammation [24,25]. Another critical mechanism involves the inhibition of the JAK/STAT signaling cascade, particularly the suppression of STAT3 phosphorylation, which results in reduced IL-6 expression and modulation of immune responses [26]. *W. somnifera* also exerts cardioprotective effects through Nrf2 cascade activation and the upregulation of Th1 cytokines, improving immune and stress responses [27].

Furthermore, *W. somnifera* affects the mitogen-activated protein kinase (MAPK) pathways by modulating p38, ERK, and JNK signaling, thereby disrupting inflammatory gene expression cascades. The plant also suppresses the production of nitric oxide (NO) and prostaglandins by downregulating iNOS and COX-2, leading to reduced tissue damage and edema in inflammatory conditions [22].

All of these mechanisms have been validated both in vitro and in vivo models, positioning *W. somnifera* as a promising phytotherapeutic agent for the treatment of chronic inflammatory diseases such as arthritis, colitis, and neuroinflammatory disorders [28].

#### 2.1.3. Neuroprotective Effects of *W. somnifera*: Molecular Mechanisms and Therapeutic Potential in Neurodegenerative Disorders

*W. somnifera* has garnered significant scientific attention for its neuroprotective properties, particularly in the context of neurodegenerative diseases such as Alzheimer’s disease (AD), Parkinson’s disease, and Huntington’s disease [29]. Its neuroprotective actions are attributed to a wide spectrum of phytochemicals which exert pleiotropic effects on neuronal function and survival. One of the primary mechanisms involves the enhancement in antioxidant defenses through the activation of the Nrf2 pathway, as already mentioned in the previous paragraph [30], as the increment in antioxidant defenses plays a crucial role in mitigating oxidative stress, a key pathological feature in neurodegenerative disorders.

In addition, *W. somnifera* extracts and isolated withanolides and withanosides can intervene in multiple AD-related pathways, enhancing in vitro axon or dendrite outgrowth [31].

Withanolide-rich extracts might favor Aβ clearance, improve Aβ-induced oxidative stress, and counteract neuronal apoptosis. The upregulation of insulin-degrading enzyme (IDE) and antioxidant defenses (e.g., heme-oxygenase-1) plays a crucial role in achieving these ameliorating effects [32]. Interestingly, in a preclinical model of AD, withaferin A reduced tau protein aggregation and amyloid oligomer formation properties by engaging chaperone proteins, such as Heat shock protein 70 and 90 (Hsp70/Hsp90, respectively) [33]. In addition, this same compound has been shown to increase α-secretase while decreasing β-secretase activity, shifting APP processing away from Aβ generation [34].

Both the plant extract and withanolide A have been shown to support cholinergic function by inhibiting acetylcholinesterase and enhancing choline acetyltransferase activity, thereby increasing synaptic levels of acetylcholine without affecting Gamma-aminobutyric acid (GABA), N-methyl-D-aspartate (NMDA), or benzodiazepine receptors [35].

Notably, both *W. somnifera* extract and withanolide A have demonstrated neuroprotective effects in hippocampal cells under hypoxic conditions, primarily through the glutathione (GSH) pathway activation [32]. Furthermore, withanolide A administration has been shown to have regenerating axonal properties and to promote synaptic repair in cortical neurons exposed to Aβ peptide [36].

*W. somnifera* also enhances cholinergic neurotransmission by inhibiting acetylcholinesterase (AChE) and increasing choline acetyltransferase (ChAT) activity, thus preserving acetylcholine levels and improving cognitive function. It also modulates synaptic plasticity by promoting dendritic and axonal regeneration, evidenced by increased neurite outgrowth in both in vitro and in vivo models [29].

Overall, the neuroprotective efficacy of *W. somnifera* is rooted in its multi-target action across oxidative, inflammatory, apoptotic, and protein aggregation pathways, offering promising therapeutic potential for the management and prevention of neurodegenerative diseases.

### 2.2. Cordyceps spp. as a Source of Bioactive Compounds in Inflammation, Cancer, and Neurodegeneration

A noteworthy herbal remedy in traditional Chinese and Tibetan medicine is the entomopathogenic fungal genus Cordyceps, which includes over 750 recognized species. Members of this fungi genus manage to infect hosts from multiple insects, arthropods, or even other fungi. They are predominantly found in humid temperate and tropical forests across the Northern Hemisphere, but are especially abundant in South, East, and Southeast Asia. Among these, 35 species have gained significant attention due to their notable pharmacological properties, including anti-cancer, antioxidant, anti-aging, and immunostimulatory effects. Cordyceps sinensis (*C. sinensis*), for example, is praised as a kidney-nourishing, lung-invigorating tonic, and together with Cordyceps militaris (*C. militaris*), their consumption is associated with vitality and energy-enhancing outcomes [37].

**Figure 2:**
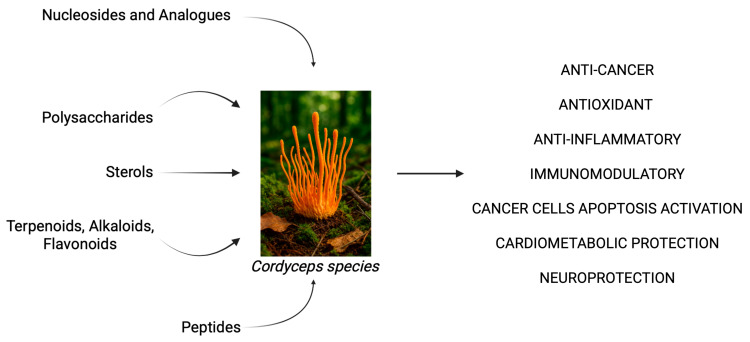
The bioactive constituents and major biological effects of *C.* spp. extracts. The scheme shows the principal bioactive compounds present in *C.* spp. (on the **left**) and the principal cellular effects (on the **right**). The figure was created in BioRender. Gabriele Tancreda. (2025) https://app.biorender.com/illustrations/681b7b959d4609b88c5f3859 (accessed on 2 June 2025).

Over 200 secondary metabolites have been isolated from *C.* spp., showcasing remarkable phytochemical diversity. Key bioactive classes are represented by nucleosides and analogs, polysaccharides, sterols, terpenoids, alkaloids, peptides, and flavonoids. Glucan and acid polysaccharides exert strong free-radical scavenging actions as well as a protective role towards mitochondrial function (Figure 2). Furthermore, numerous compounds belonging to the sterols, triterpenoids, and peptides group (e.g., cordymin) have been studied for their anti-inflammatory activity via the inhibition of the NF-κB pathway and the suppression of pro-inflammatory cytokines such as tumor necrosis factor alpha (TNF-α) and IL-1β [38].

Several in vitro studies have highlighted the immunostimulatory and immunomodulatory effects of *C.* spp. In murine macrophage line cell RAW 264.7, derived from mice infected with Abelson murine leukemia virus, a high-molecular-weight polysaccharide isolated from *C. militaris* demonstrated anti-inflammatory, cytokine induction and macrophage activation properties [39].

Another pivotal bioactive compound found in *C.* spp., cordycepin, displays broad anti-inflammatory, antioxidant, anti-apoptotic, chemopreventive, and immunomodulatory properties [38]. For example, in human colon cancer cells HT-29, cordycepin features apoptosis activation properties through the Death receptor 3 (DR3) pathway [40].

Furthermore, both cordycepin and *C. militaris* extracts have been associated with cancer-sensitizing and immunomodulatory actions in cancer cell models, altering the expression of markers involved in immune-mediated killing of cancer cells, death receptors such as Fas receptor (FasR) and Death receptor 4 (DR4), and apoptosis pathways [41].

In the context of cardiometabolic disease, the in vivo administration of a polysaccharide component isolated from *Cordyceps cicadae (C. cicadae)* was linked to improvements in mucosal barrier indicators, lower levels of serum lipopolysaccharide (LPS), and microbiota restoration properties in diabetic mice [42].

Among the primary biological effects associated with *C. sinensis* extract are its neuroprotective and cognitive-enhancing properties, mediated through the activation of antioxidant signaling pathways. A polysaccharide isolated from *C. sinensis* has demonstrated protective effects against hydrogen peroxide-induced damage in PC12 neural cell models, evidenced by reduced malondialdehyde (MDA) levels and increased activities of antioxidant enzymes such as glutathione peroxidase (GPx) and superoxide dismutase (SOD) in pretreated samples [43]. Using the same in vitro model (PC12 cells), Shen et al. confirmed the role of an acid polysaccharide derived from *C. mycelia* in increasing cellular survival and antioxidant enzyme levels post oxidative damage exposure. The study also pointed out that lactate dehydrogenase and MDA levels decrease [44].

Regenerative properties have been found in HT-29 cells and indomethacin-injured rats. In fact, hot water extract of *C. sinensis* stimulated cell proliferation and accelerated gastric mucosal healing [45].

## 3. Emerging Role of Phytotherapeutic Compounds from *W. somnifera* and *C.* spp. In Alzheimer’s Disease

### 3.1. Neuroprotective Properties of W. somnifera Extract and Bioactive Constituents in Preclinical Model of Alzheimer’s Disease

Among the 33 studies that emerged from electronic databases (18—Google Scholar; 15—PubMed), only 11 were considered eligible for inclusion in the present review, as we excluded duplicates and review articles, focusing our attention solely on studies that reported experimental data on the effects of *W. somnifera* on AD (for further details, please refer to Supplementary Figure S1, which outlines the screening process and the exclusion criteria applied). As shown in Table 1, both methanolic and aqueous extracts of *W. somnifera* effectively promoted neuronal health via the upregulation of antioxidant enzymes and the stimulation of Aβ clearance in preclinical rodent models of AD. High-dose treatments were found not only to be safe and well tolerated but also to exert dose-dependent protective effects. Interestingly, cognitive and behavioral improvements, preservation of brain structure, and inhibition of Aβ deposition were also reported for the bioactive constituents withanone and withanoside IV when administered individually.

*W. somnifera* and its bioactive components, withanone and withanoside IV, have been studied for their neuroprotective and neuroenhancing properties in vitro and in preclinical models. The antioxidant activity is achieved via Nrf2 pathway activation and ROS scavenging [53].

As reported by Gladen-Skolarsky et al., administration of an aqueous extract of *W. somnifera* in transgenic mice mimicking AD determined the upregulation of the genes Nrf2 and NAD(P)H quinone oxidoreductase 1 (NQO1) in the cortex of mouse brain, along with the concomitant reduction in Aβ plaque deposition in the hippocampus and improvements in cognitive, behavioral, and psychological symptoms [46].

Likewise, mice treated with a methanolic extract of *W. somnifera* demonstrated positive modulation of oxidative stress and antioxidant activity through the reduction in MDA levels and the upregulation of GSH and SOD levels analyzed in the brain [47].

Interestingly, Afewerky et al. reported a significant role of this phytotherapeutic in promoting mitochondrial health and function. Specifically, upregulation of the neuronal sodium–calcium exchanger isoform 3 (NCX3) in the cortex and hippocampus with the use of the herb’s methanolic extract is suggested to exert a protective effect against Ca^2^⁺ dyshomeostasis [48].

A pivotal anti-Alzheimer mechanism reported for *W. somnifera* extract is Aβ formation inhibition in the brains of treated animal models. This remarkable result may be attributed to the combined effects exerted by this plant, primarily its antioxidant and anti-inflammatory activity, as well as the documented suppressing activity of withanone towards β- and γ-secretase levels in the brains of mice. Β-secretase is the aspartic enzyme responsible for the cleavage of APP and the generation of the C99 peptide fragment that is subsequently processed by γ-secretase, leading to the formation of Aβ40 and Aβ42. By inhibiting the enzymatic processing cascade, withanone-rich *W. somnifera* extracts might be a promising formulation for managing AD clinical features [53].

Sehgal et al. identified a novel mechanism by which *W. somnifera* promotes Aβ clearance. They showed that high-dose extract administration markedly increases hepatocyte expression of the low-density lipoprotein receptor-related protein (LRP), the primary receptor mediating Aβ endocytosis. Moreover, these hepatocytes shed LRP’s large extracellular domain into the bloodstream as soluble LRP (sLRP), which binds circulating Aβ and traps it in the periphery, thereby preventing its re-entry into the brain [49].

Furthermore, as described by Abdullah et al., the pharmacological potential of *W. somnifera* also includes support for cholinergic function through the downregulation of brain levels of AChE and BACE, both of which are involved in acetylcholine degradation [51].

Another interesting target reported for this medical plant is glutamate dehydrogenase. Methanolic and aqueous extracts of *W. somnifera* decreased glutamate expression in the cortex and hippocampus of treated rodents to levels comparable to the control group in scopolamine-induced Wistar albino male rats. Visweswari et al. were able to link the upregulation of glutamate dehydrogenase activity in the brain to the attenuation of neural cell loss in the cortex and hippocampus via histopathological observations [50]. This further testifies to the positive impact of *W. somnifera* extract and its bioactive compounds on neurotoxicity and the recovery of cognitive function, learning, memory, and behavioral deficits, all recognized as hallmarks of AD clinical features [54,55].

Despite the lack of randomized clinical trials exploring the anti-Alzheimer effects of *W. somnifera* extracts in affected patients, Lopresti et al. showed that 240 mg of a standardized ashwagandha extract once daily for 60 days exerted anxiolytic and mood-enhancing effects in adults with self-reported high stress [56]. Likewise, the results from the 8-week randomized clinical trial carried out by Pandit et al. confirmed the previous ones. The three administered doses of standardized *W. somnifera* extracts produced significant, dose-dependent drops in fasting plasma cortisol and adrenocorticotropic hormone, lowering perceived stress and improving anxiety and depressive scores. Remarkably, the treatment led to a significant drop in pro-inflammatory cytokines too, suggesting protection against oxidative stress [57].

### 3.2. Neuroprotective Properties of C. spp. Extract and Bioactive Constituents in Preclinical Model of Alzheimer’s Disease

The number of studies regarding the neuroprotective mechanisms and pharmacological application of *C.* spp. is more limited than those explored for *W. somnifera*. In detail, 10 studies published in the last 20 years emerged in the electronic database (4 from Google Scholar and 6 from PubMed). Of these, only four studies (two from Google Scholar and two from PubMed) were included in this review, as they reported experimental data on the effects of *C.* spp. on AD (additional information regarding the screening methodology and exclusion criteria is provided in Supplementary Figure S2). *C.* spp. (i.e., *militaris*, *cicadeae*, *ophioglossoides mycelium)* and the bioactive constituent cordycepin, administered alone, were associated with improvements in cognitive and memory skills, Aβ deposition, and key enzyme activities in preclinical models of AD (Table 2).

In the context of AD, both *C.* spp. extracts and cordycepin alone show a promising role in the management of cognitive dysfunction, neuroinflammation, and Aβ plaque deposition in preclinical models of this neurodegenerative disease. Consistently, in a rodent model of Parkinson’s disease, cordycepin treatment preserved mitochondrial morphology and function, preventing vacuolar degeneration and rotenone-induced neurotoxicity [62].

In another study, cordycepin exerted a pivotal role in preserving the integrity of white matter in the brain of a traumatic brain injury mouse model and ameliorated neurological deficits. The adenosine analog conferred long-term neuroprotection via the microglia and macrophage inhibition, the promotion of anti-inflammatory pathways, and the suppression of matrix metalloprotease (MMP) activity, together with neutrophil infiltration [63].

Wu et al. observed that the neuroprotective properties of *C.* spp. can be greatly influenced by the culture conditions. In their study, co-fermentation of *C. cicadea* in deep ocean water boosted the fungi’s mycelial biomass and its N(6)-(2-Hydroxyethyl) adenosine (HEA) content. The novel multi-component fungal preparation effectively inhibited the amyloidogenic pathway, reducing the protein expression of β-secretase and Aβ40 in the hippocampus of rats. It also prevented tau hyperphosphorylation and suppressed microglial inflammation by upregulating the soluble receptors for the advanced glycation end products (sRAGE) and downregulating TNF-α, IL-1β, and IL-6, ultimately leading to ameliorating results in AD-like cognitive deficits. One of the mechanisms underlying these results involves the upregulation of magnesium transporter 1 (MAGT1), a protein that facilitates Mg^2^⁺ influx into neurons and supports endothelial barrier function. Co-fermentation with deep ocean water enables *C. cicadae* to absorb and biotransform Mg^2^⁺ into an organic form. Elevated intracellular Mg^2^⁺ levels suppress BACE expression and inhibit GSK-3β activity, thereby reducing tau generation and hyperphosphorylation and limiting Aβ production [59].

Interestingly, in the study carried out by Ock Kim et al., the treatment with cordycepin from *C. militaris* (10 mg/kg body weight/day for 4 weeks) improved markers of cellular senescence and enhanced neuronal plasticity, evaluated through the upregulation of microtubule-associated protein 2 (MAP2) and neuronal nuclei (NeuN). Furthermore, as previously pointed out, cordycepin skewed microglial polarization from the M1 pro-inflammatory pathway towards the anti-inflammatory one, promoting the release of neurotrophic factors and the creation of a neuroprotective microenvironment, dampening chronic inflammation that drives Aβ deposition and tau pathology [60].

Consistent with the evidence discussed, the involvement of these pleiotropic mechanisms is suggested to contribute to the improvement in pathological features of AD and to support the restoration of cognitive and behavioral functions, including spatial memory and learning capacity, as reported by Jin et al. and He et al. [58,61].

To the best of our knowledge, there is a lack of scientific research concerning clinical trials that explore the effects of *C.* spp. extracts in ameliorating cognitive and psychological symptoms in human cohorts. Nonetheless, coherently with in vitro and preclinical evidence, the administration of *C. mycelium* culture extract for 8 weeks to healthy adults resulted in significant enhancements in natural killer (NK) cell cytotoxic activity compared to the placebo group, suggesting possible clinical translation regarding AD [64].

## 4. Conclusions

Based on the current literature, *W. somnifera* and *C.* spp. extracts may represent valuable adjuncts to established pharmacological treatments such as donepezil. In addition to compelling preclinical data, randomized placebo-controlled clinical trials have reported improvements in multiple parameters directly involved in the onset and progression of Alzheimer’s disease while also confirming the in vivo safety and tolerability of these natural compounds. Their neuroprotective properties—spanning antioxidant and anti-inflammatory effects, the modulation of β-amyloid metabolism, the inhibition of amyloidogenic enzymes, mitochondrial support, and the enhancement in neuronal plasticity—complement the cholinesterase inhibitory action of donepezil. The combination of these mechanisms may offer a broader and more integrated therapeutic approach to mitigating the neurodegenerative and neuroinflammatory processes underlying Alzheimer’s pathology. Notably, bioactive constituents such as withanolides (e.g., withanone and withanoside IV) and cordycepin appear central to these effects.

However, translating findings from in vitro and in vivo studies in murine models to clinical applications in humans presents several limitations, including the pharmacokinetic and pharmacodynamic behavior of the compounds in humans, which often differs from that observed in animal models. In this specific case, it is necessary to consider that when compounds are administered orally, their ability to reach the CNS is frequently hindered by poor bioavailability and limited permeability across the blood–brain barrier (BBB). Therefore, further high-quality clinical studies and pharmacological optimization are necessary before bioactive compounds from *W. somnifera* and *C.* spp. can be considered co-adjuvants in the comprehensive management of AD.

## Figures and Tables

**Table 1:** Summary of included studies using aqueous/methanolic extract of *W. somnifera* or its bioactive constituents in preclinical models of Alzheimer’s Disease.

Num	Year	Ref. Country	Paper Title	Animal Model (Species; Gender)	Intervention	Control	Results
1	2024	[46]; USA and Canada	*W. somnifera* (ashwagandha) improves spatial memory, anxiety, and depressive-like behavior in the 5× FAD mouse model of Alzheimer’s disease.	Mice (5× FAD; male and female)	Administration of aqueous extract of dried *W. somnifera* at 0, 0.5, or 2.5 mg/mL via drinking water ad libitum for 4 weeks. Behavioral testing was conducted in the final week of treatment.	Wild-type mice administered drinking water	▪ The aqueous extract of *W. somnifera* improved spatial memory, anxiety-related symptoms, and depressive-like behavior at both intervention doses. ▪ It reduced deposition of Aβ plaques in the cortex and hippocampus in the 2.5 mg/mL intervention group compared to the 0 mg/mL group. ▪ The aqueous extract of *W. somnifera* reduced astrocytic and microglial activation through the modulation of GFAP in the cortex and hippocampus and IBA1 in the hippocampus. ▪ The aqueous extract of *W. somnifera* induced an increase in the antioxidant response in the cortex through upregulation of genes Nrf2 and NQO1.
2	2014	[47]; India	*W. somnifera* and Eclipta alba ameliorate oxidative stress-induced mitochondrial dysfunction in an animal model of Alzheimer’s disease.	Albino rats (Wistar; male)	60 rats were divided into ten groups: a control group, a toxicant group (carboxymethyl cellulose), two standard drug-treated groups (donepezil and Piracetam), three groups treated with a methanolic extract of *W. somnifera* (at doses of 50, 100, and 200 mg/kg), and three groups treated with methanolic extract of Eclipta alba (at doses of 50, 100, and 200 mg/kg). The intervention lasted for 8 days. On the 8th day, animals were subjected to scopolamine treatment and induction of oxidative stress.	Albino rats administered carboxymethyl cellulose	▪ Pretreatment with the methanolic extract of *W. somnifera* successfully reversed scopolamine-induced amnesia in a dose-dependent manner. ▪ Pretreatment with the methanolic extract of *W. somnifera* decreased the concentration of MDA measured in brain homogenates. ▪ Pretreatment with the methanolic extract of *W. somnifera* at a dose of 200 mg/mL successfully prevented mitochondrial functional reduction.
3	2022	[48]; China	Sodium–calcium exchanger isoform-3-targeted *W. somnifera* (L.) Dunal therapeutic intervention ameliorates cognition in the 5× FAD mouse model of Alzheimer’s disease.	Mice (5× FAD; male)	Animals were divided into four groups: control group (1 mL of saline 0.9%/day), low-dose treatment group (200 mg/kg/day of methanol extract of *W. somnifera*), high-dose treatment group (400 mg/kg/day of methanol extract of *W. somnifera*), and a positive control group (200 mg/kg day of resveratrol).	5× FAD mice treated with 1 mL of saline 0.9%	▪ Learning performance, memory recall, spatial learning, and working memory improved in mice treated with W. somnifera at both doses. ▪ *W. somnifera* at lower and higher doses upregulated NC3X expression in the cortex and hippocampus, suggesting a positive effect on the calcium-regulated mechanisms in neurons. ▪ Both doses of *W. somnifera* reduced total Aβ levels in the cortex and hippocampus. ▪ Lower and higher doses of *W. somnifera* reduced the deposition of Aβ plaques in the cortex and hippocampus. ▪ *W. somnifera* positively impacted oxidative stress and antioxidant activity through the regulation of MDA, SOD, and GHS brain levels.
4	2012	[49]; India	*W. somnifera* reverses Alzheimer’s disease pathology by enhancing low-density lipoprotein receptor-related protein in liver.	Mice (APP/PS1, APPSwInd (J20 line), WT; male and female)	Transgenic mice were divided into two groups: one receiving *W. somnifera*’s extract (1 g/kg) in ethanol and the control group receiving an equivalent volume of ethanol daily via oral administration for 7–30 days.	Transgenic mice administered drinking water	▪ Withania extract administration led to complete recovery of the behavioral deficits in middle-aged APP/PS1 and APPSwInd J20 mice evaluated through the radial maze task and Morris water maze. Gradual improvements were also observed in the old APP/PS1 mice group. ▪ Withania extract administration reduced amyloid pathology, favoring the elimination of plaques in the cortex and hippocampus in middle-aged APP/PS1 mice and markedly reducing plaque burden in old APP/PS1 and APPSwInd J20 mice. Furthermore, the treatment determined an important reduction in Aβ deposition in brain microvessels in both old APP/PS1 and APPSwInd J20 mice. ▪ *W. somnifera* administration significantly decreased Aβ42 in both the cortex and hippocampus in middle-aged male and old APP/PS1 mice without affecting APP mRNA or protein expression. A significant decrease in Aβ oligomer levels in the cortex of APP/PS1 mice after the 30-day treatment was observed. ▪ Between days 7 and 14, treatment groups presented a significant increase in plasma Aβ 42/40 levels, suggesting the protein’s clearance from the brain to the bloodstream. ▪ LPR mRNA and protein expression in the cortex increased significantly between days 14 and 30 of treatment. ▪ A progressive increase in liver NEP and LRP mRNA and levels of plasma sLRP after day 7 of treatment was observed.
5	2021	[50]; India	*W. somnifera* against glutamate excitotoxicity and neuronal cell loss in a scopolamine-induced rat model of Alzheimer’s disease.	Rats (Wistar albino; male)	Rats were divided into five groups: control; scopolamine-induced Alzheimer’s model (2 mg/kg intraperitoneally) + normal saline administered orally; scopolamine-induced Alzheimer’s model + donepezil hydrochloride (5 mg/kg) administered orally. The intervention lasted for 30 days. Rats were then decapitated and their brains removed for analysis.	Scopolamine-induced Alzheimer’s model of Wistar albino male rats	▪ Methanolic and water *W. somnifera* extracts decreased glutamate expression in the cortex and hippocampus to levels comparable to the control group. ▪ The *W. somnifera* extract induced upregulation in glutamate dehydrogenase activity in the cortex and hippocampus comparable to the control group. ▪ Histopathological studies highlighted the *W. somnifera* extract’s’ role in reversing neuronal cell loss in the cortex and hippocampus of treated groups compared to the non-treated Alzheimer’s model.
6	2022	[51]; Saudi Arabi	The neuroprotective effect of Lycopodium and *W. somnifera* on Alzheimer’s disease induced by aluminum chloride in rats.	Rats (Wistar albino; female)	Rats were divided into five groups: the control administered with distilled water; the AD group (aluminum chloride 175 mg/kg) treated with a water extract of Lycopodium (50 mg/kg); the AD group (aluminum chloride 175 mg/kg) treated with a water extract of *W. somnifera* (200 mg/kg); the AD group (aluminum chloride 175 mg/kg) treated with *W. somnifera* (200 mg/kg) and Lycopodium extract (50 mg/kg).	Aluminum chloride-induced Alzheimer’s model of Wistar albino female rats	▪ *W. somnifera* administration alone or combined with Lycopodium exerted beneficial effects towards cholinergic enzymes through the downregulation of AChE and BchE expressed in brain homogenates. ▪ *W. somnifera* administration alone or combined with Lycopodium reduced Aβ-42 concentration in brain homogenates. ▪ *W. somnifera* administration alone or combined with Lycopodium positively impacted the levels of tyrosine hydroxylase and the activity of Na^+^/K^+^ ATPase in the brain homogenates or rats.
7	2022	[48]; China	Aβ_1–42_-related sodium–calcium exchanger isoform-3 downregulation in models of Alzheimer’s disease: therapeutic significance of *W. somnifera* root extract.	Mice (5× FAD; male and female)	5× FAD mice were administered 300 mg/kg/day of *W. somnifera*’s methanolic extract via oral gavage for 30 days.	5× FAD mice administered with vehicle	▪ *W. somnifera* treatment ameliorated cognitive and behavioral parameters evaluated through the Barnes circular maze, time spent in the target zone, and Y-maze task. ▪ *W. somnifera* treatment increased the protein expression of NCX3 in the cortex and hippocampus of brain homogenates. ▪ *W. somnifera* treatment attenuated the deposition of Aβ-42 and plaque formation in the cortex and hippocampus of brain homogenates. ▪ *W. somnifera* treatment positively modulated oxidative stress in the mice’s brain, inducing the reduction in MDA concentration and upregulating SOD and GSH levels.
8	2014	[52]; India	Dose-dependent effect of *W. somnifera* on the cholinergic system in scopolamine-induced Alzheimer’s disease in rats.	Rats (Wistar; male)	Rats were divided into groups: a normal control; a scopolamine-induced Alzheimer’s control (2 mg/kg per b.w. intraperitoneally); and experimental groups treated with methanol or aqueous extracts (100, 200, 300 mg/kg, orally) or donepezil (5 mg/kg, orally). Treatments lasted 10 days to evaluate their potential neuroprotective effects against scopolamine-induced cognitive impairment.	Scopolamine-induced Alzheimer’s male Wistar rats	▪ Dose-dependent decreases in the activity of AChE in the cerebral cortex, cerebellum, and hippocampus but not the hypothalamus in rats treated with the methanolic extract of *W. somnifera* (300 mg/kg b.w.) were observed. ▪ The aqueous extract of *W. somnifera* (300 mg/kg b.w.) induced a dose-dependent decrease in AChE activity in all brain regions analyzed. ▪ The methanol extract of *W. somnifera* at 300 mg/kg significantly increased the concentration of Ach in the hippocampus of rat brains. ▪ The aqueous extract of *W. somnifera* at 300 mg/kg increased the concentration of Ach in the hippocampus and cerebellum of rat brains.
9	2018	[53]; India	Multifunctional neuroprotective effect of withanone, a compound from *W. somnifera* roots, in alleviating cognitive dysfunction.	Rats (Wistar; male)	Rats were divided into six groups: sham control; CSF control group (bilateral ICV injection 10 μL on each side); STZ control group (ICV injection of STZ 10 μL on each side); three drug-treated groups (5 or 10 or 20 mg/kg of withanone, orally). The drug treatment lasted for 21 days after the surgery.	Rats in the STZ control group (ICV injection of STZ 10 μL on each side)	▪ Withanone (20 mg/kg) led to a significant decrease in transfer latency, indicating improvements in cognitive function in treated STZ rats. ▪ Withanone (20 mg/kg) reduced brain Aβ deposition and concentration. ▪ Withanone (5, 10, and 20 mg/kg) significantly decreased the expression of TNF-α, IL-1β, IL-6, and MCP-1 in the brain of treated rats in a dose-dependent manner. ▪ Withanone (10 and 20 mg/kg) significantly increased Ach levels in the brain of treated rats. ▪ Withanone (10 and 20 mg/kg) decreased the levels of β- and γ-secretase enzymes in the brains of treated rats. ▪ Withanone (5, 10, and 20 mg kg) improved levels of oxidative stress parameters (MDA, nitric oxide, catalase) in brain tissue of treated rats in a dose-dependent manner. ▪ Withanone (20 mg/kg) modulated the expression of Th1 and Th17 cytokines in the peripheral blood.
10	2006	[54]; Japan	Withanoside IV and its active metabolite, sominone, attenuate Aβ(25–35)-induced neurodegeneration.	Mice (ddY; male)	Mice were divided into two groups: the treatment group (25 nmol of Aβ(25–35) injected into the right ventricle); and the control group (vehicle injected into the right ventricle). Seven days after intra-cerebrovascular injection, the treatment group received withanoside IV (10 µmol/kg/day) administered once daily for 13 days. The vehicle group received 0.5% Arabic gum solution administered orally on the same schedule.	Mice in the vehicle group were administered with 0.5% Arabic gum solution after intra-cerebrovascular injection of Aβ(25–35)	▪ Withanoside IV treatment led to improvements in learning acquisition and memory retention. Locomotor abilities were found to be similar between groups. ▪ Withanone IV treatment increased the NF-H positive area in the temporal cortex of treated mice. Furthermore, the treatment increased the MAP-2 positive area in region C3 of the hippocampus and in the parietal cortex and synaptophysin-positive area in the hippocampal CA1 region, parietal, and temporal cortexes.
11	2021	[55]; India	Development of synergy-based combination for learning and memory using in vitro, in vivo, and TLC-MS-Bioautographic studies.	Rats (Wistar; male)	Rats were divided into nine groups: control group (10 mL 0.5% CMC/rat); negative control (scopolamine 2 mg/kg); treatment groups 3 to 6 received either the selected high or low dose of an aqueous extract of *W. somnifera* or *Myristica fragans* together with scopolamine; treatment group 7 was administered with the high dose of the *W. somnifera* extract in combination with the low dose of Myristica *fragans* together with scopolamine; treatment group 8 was administered with the high dose of Myristica *fragans* in combination with the low dose of *W. somnifera* together with scopolamine; the positive control group (Pyracetam 200 mg/kg) received scopolamine. The treatment lasted for 37 days, and behavioral tests were carried out between days 30 and 36.	Wistar male rats were administered with scopolamine	▪ Treatment with *W. somnifera* extract alone or in combination with Myristica fragrans led to marked improvements in behavioral and cognitive function in Alzheimer’s disease rat models. The most significant results were obtained with the coadministration of the high dose of *W. somnifera* in combination with the low dose of Myristica *fragrans*.

**Table 2:** Summary of included studies using aqueous/ethanolic/methanolic extract of *C.* spp. or its bioactive constituents in preclinical models of Alzheimer’s disease.

Num	Year	Ref. Country	Paper Title	Animal Model (Specie; Gender)	Experimental Design and Intervention	Control	Results
1	2018	[58]; Korea	Protective role of *C. s militaris* in in vivo Aβ_1–42_-induced Alzheimer’s disease.	Mice (IR; male)	Mice were divided into the following groups: normal group (water; Aβ1–42 intra-cerebrovascularly injected control group (3 μL/3 min); Aβ1–42 injection + ethanol extract of *C. militaris* (100 mg/kg/day) group; Aβ1–42 injections + *C.* mimlitaris ethanol extract (200 mg/kg/day) group; Aβ1–42 injections + donepezil (5 mg/kg/day) group. Aβ1–42 injections took place on day 3 of the experimental schedule, while the oral administration of *C.* spp. extract or donepezil started on day 8 and continued till day 23. Behavioral and cognitive functions were evaluated between days 15 and 23. Sacrifices took place on day 24 of the experiment.	Male IRC mice with Aβ1–42 intra-cerebrovascularly injected (3 μL/3 min)	*C. militaris* extract at 100 and 200 mg/kg/day led to significant improvements in cognitive and behavioral functions in treated mice. Space perceptive ability, objective cognitive ability, and cognitive abilities related to the Morris water maze test all improved in relation to the treatment.
2	2021	[59]; Taiwan	*C. cicadae* NTTU 868 mycelium with the addition of bioavailable forms of magnesium from deep ocean water prevents Aβ40 and Streptozotocin-induced memory deficit by suppressing Alzheimer’s disease risk factors and increasing magnesium uptake in the brain.	Rats (Sprague Dawley; male)	Rats were divided into the following groups: normal control (standard chow); vehicle group (intra-cerebrovascular injection of the vehicle); negative control injected with Aβ-STZ solution (24.299 μg Aβ40 + 0.9 mg STZ in 180 μL) in the left lateral ventricle; treatment group administered with Aβ-STZ solution + deep ocean water (0.0976 mL/kg/day); treatment groups administered with Aβ-STZ solution + *C. cicadae* cultured in ultrapure water (200 mg/kg/day) or deep ocean water (220 mg/kg/day) or in MgCl₂ (137 mg/kg day); treatment groups administered with N6-(2-hydroxyethyl)-adenosine (1.12 mg/kg/day) or polysaccharides (1.5 mg/kg/day). The treatment lasted for 28 days after the intra-cerebrovascular injection, and experiments were conducted during the last week before sacrifice.	The negative control group was injected intra-cerebrovascularly with Aβ-STZ solution (24.299 μg Aβ40 + 0.9 mg STZ in 180 μL) in the left lateral ventricle	▪ Learning and memory skills improved the most in the group administered with *C. cicadae* cultured in deep ocean water. ▪ Cultured *C. cicadae* in deep ocean water led to significant decreases in Aβ40 and BACE protein concentrations in the hippocampus of rats. ▪ The administration of *C. cicadae* cultured in deep ocean water was the most effective in decreasing the protein expression of pro-inflammatory in the hippocampus and cortex. Other beneficial treatments, although less effective, included *C. cicadae* cultured under different experimental conditions and the administration of polysaccharides and N6-(2-hydroxyethyl)-adenosine. ▪ sRAGE was upregulated the most in the group administered with *C. cicadae* cultured in deep ocean water. ▪ *C. cicadae* cultured in deep ocean water led to increased concentrations of Mg^2+^ in the hippocampus and cortex. ▪ Groups administered with deep ocean water, *C. cicadae* cultured in deep ocean water, and magnesium demonstrated a higher concentration of the MAGT1 protein in the hippocampus.
3	2023	[60]; China	Cordycepin improved neuronal synaptic plasticity through CREB-induced NGF upregulation driven by MG-M2 polarization, a microglia-neuron symphony in AD.	Mice (double-transgenic APP/PS1, C56BL/6; male and female)	Double-transgenic APP/PS1 mice were divided into two groups: the negative control group was administered intragastrically with distilled water; the treated group was administered with q water extract of cordycepin from *C. militaris* (10 mg/kg/day). C56BL/6 mice served as the positive control group, administered intragastrically with distilled water. The treatment lasted 4 weeks.	Double-transgenic APP/PS1 mice	▪ Cordycepin treatment resulted in improvements in learning and memory impairments in APP/PS1 mice while maintaining the same locomotor capacities. ▪ Cordycepin treatment led to reduced cellular senescence through the downregulation of β-Galactosidase’s expression in the hippocampus. ▪ Cordycepin treatment resulted in the enhancement in neuronal plasticity through the upregulation of neuronal markers MAP2 and NeuN. Furthermore, cordycepin administration led to the upregulation of synaptic proteins GAP43, synaptophysin, and PSD-95. ▪ Cordycepin treatment resulted in improved synaptic morphology observed via transmission electron microscopy. ▪ Cordycepin administration led to a remarkable decrease in pro-inflammatory markers (IL-1β, TNF-α, iNOS) and an increase in anti-inflammatory markers (IL-10, TGF-β, Arg1) in the hippocampus. Furthermore, cordycepin administration resulted in a decrease in Iba-1 and CD86 expression while increasing the expression of CD206 in the hippocampus of APP/PS1 mice. ▪ Cordycepin treatment resulted in the restoration of NGF concentrations in the hippocampus of APP/PS1 mice to levels comparable to those of C56BL/6 mice. Furthermore, cordycepin treatment upregulated the expression of CREB and pCREB.
4	2004	[61]; Korea	Mycelial extract of *C.* spp. ophioglossoides prevents neuronal cell death and ameliorates b-amyloid peptide-induced memory deficits in rats	Rats (Sprague Dawley male)	Rats were divided into three different groups: the negative control group was injected intracranially with Aβ (1 mg/mL) to induce AD-like pathology; the treatment group was administered with methanolic extract of *C.* spp. ophioglossoides mycelium (100 mg/kg/day) intraperitoneally before Aβ (1 mg/mL) injection; rats in the positive control group received distilled water intracranially. The experiment lasted 3 weeks.	Rats injected intracranially with Aβ (1 mg/mL)	Treatment with the extract of *C.* spp. ophioglossoides mycelium was effective in preventing pathological alterations in spatial memory and learning capacity induced by Aβ injection.

## Data Availability

Not applicable.

## Supplementary Materials

The following supporting information can be downloaded at https://www.mdpi.com/article/10.3390/ijms26115403/s1.
