# Supplementary material for: Preclinical Evidence of Withania somnifera and Cordyceps spp.: Neuroprotective Properties for the Management of Alzheimer’s Disease
Source: Int J Mol Sci. 2025 Jun 4;26(11):5403. doi: 10.3390/ijms26115403 (PMC12156217; doi:10.3390/ijms26115403)

Supplementary Figure S1: Schematic representation of the criteria used to screen and select the publications about *W. somnifera* effects on AD.

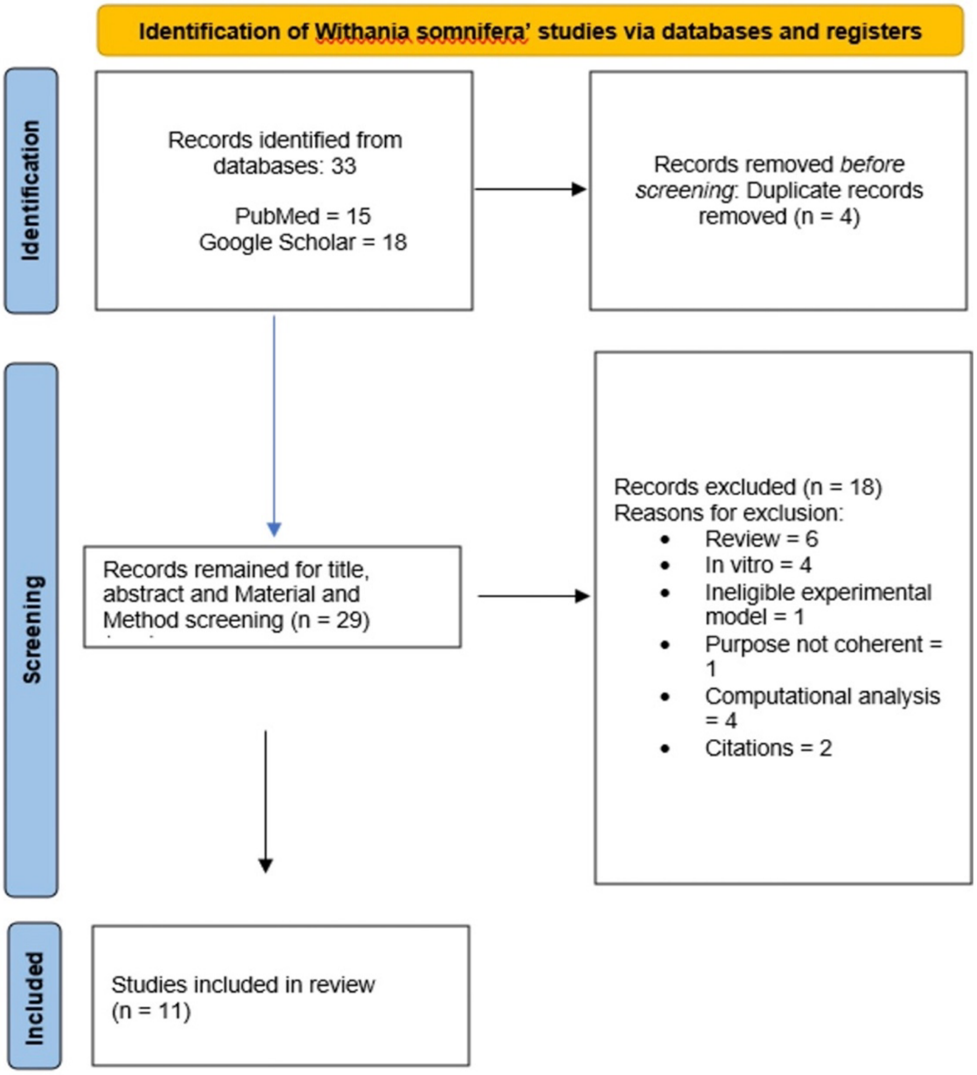

Supplementary Figure S2: Schematic representation of the criteria used to screen and select the publications about *C.spp.* effects on AD.

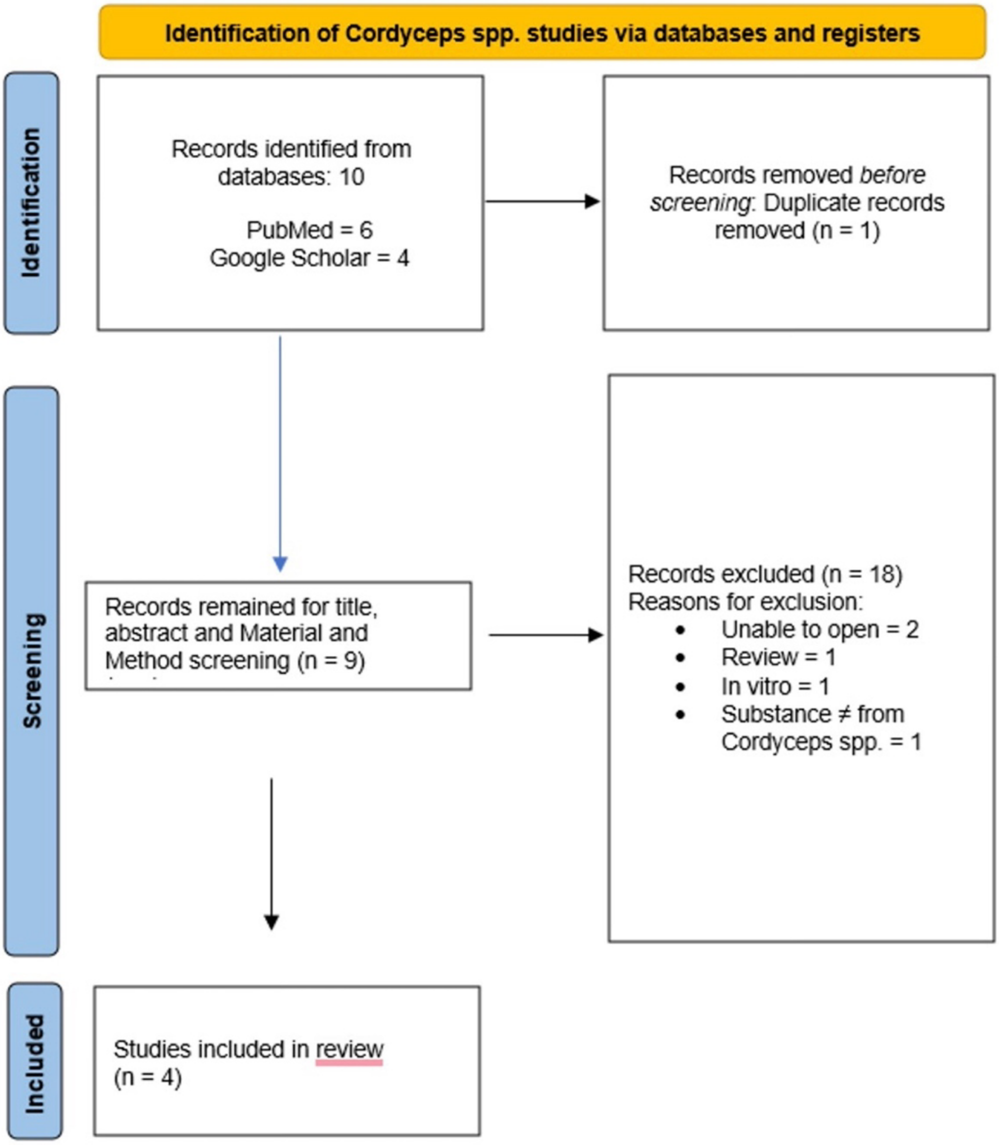

Supplement: Supplementary file 1 [file ijms-26-05403-s001.zip › ijms-3662309-supplementary.pdf]
